# Comparison of intrinsic foot muscle morphology and isometric strength among runners with different strike patterns

**DOI:** 10.1371/journal.pone.0286645

**Published:** 2023-06-02

**Authors:** Zhen Wei, Jingjing Liao, Xiaomei Hu, Pan Li, Lin Wang

**Affiliations:** Key Laboratory of Exercise and Health Sciences, Shanghai University of Sport, Shanghai, China; Universitatea de Medicina si Farmacie Victor Babes din Timisoara, ROMANIA

## Abstract

This study aimed to compare the intrinsic foot muscle (IFM) morphology and isometric strength among runners with habitual rearfoot strike (RFS) and non-rearfoot strike (NRFS) patterns. A total of 70 recreational male runners were included in this study (32 RFS and 38 NRFS), an ultrasound device and hand-held dynamometry were used to measure IFM morphology and isometric strength. Results indicated that the RFS runners had significantly thicker tibialis anterior (P = 0.01, ES = 0.64, 95% CI [0.01–0.07]) in IFMs morphology and higher Toe2345 flexion strength in IFMs strength (P = 0.04, ES = 0.50, 95% CI [0.01–0.27]) than NRFS runners, the cross-sectional area of flexor digitorum brevis was positively correlated with T2345 flexion strength (r = 0.33, p = 0.04), T12345 (r = 0.37, p = 0.02) and Doming (r = 0.36, p = 0.03) for runners with NRFS. IFMs morphology and isometric strength were associated with foot strike pattern, preliminary findings provide new perspectives for NRFS runners through the simple measurement of IFMs morphological characteristics predicting IFMs strength, future studies could adopt IFMs training to compensate the muscle strength defects and prevent foot-related injuries.

## Introduction

Running is one of the most popular and inexpensive form of recreational physical activities. Unfortunately, up to 79.3% of runners experience lower extremity musculoskeletal injuries [1]. Among the factors contributing to running-related injuries, foot strike patterns (FSPs) are essential factors which have been studied in recent years.

FSP is defined according to the location of the centre of pressure when the foot initially touches the ground. Studies have reported that 75% of FSP are rearfoot strike (RFS), 23% are midfoot strike (MFS) and 2% are forefoot strike (FFS) in long-distance runners [2]. In runners with different FSPs, ground reaction force is regulated through the active modulation of the activities of lower limb muscles during running [3, 4]. FFS runners have higher electromyographic (EMG) amplitudes of the medial and lateral gastrocnemii and lower EMG amplitude of the tibialis anterior because of the ankle in plantarflexion when the heel comes into contact with the ground [5]. RFS runners have higher EMG amplitudes of the tibialis anterior because of ankle in dorsiflexion when the heel comes into contact with the ground [6]. For the lower limbs muscle morphology and strength, Gonzales et al [3, 7] revealed that RFS runners have smaller plantar flexion moment, lower tibialis anterior echo intensity, larger tibialis anterior pennation angle and smaller lateral gastrocnemius pennation angle, they established that FSP is associated with lower limb muscle function and architecture.

However, current studies exploring the mechanism contributing to lower extremity injuries have mainly focused on thigh and calf muscles, and knowledge of the role of intrinsic foot muscles (IFMs) is limited [8, 9]. IFMs, categorised as active subsystems in the foot core system, play an important role in running activities, mainly including abductor halluces (ABH), flexor digitorum brevis (FDB), flexor hallucis brevis (FHB) and quadratus plantae (QP). IFMs originate from and are inserted on the foot and consist of four layers of muscles deep to the plantar aponeurosis and may thus act as important shock absorbers, weight support structures and locomotive effectors [8, 10] to support foot arch activity. In addition, IFMs are load dependent, synergistic and modulating, acting like a spring and providing foot stability and flexibility [11, 12].

Numerous studies that have explored significant differences in biomechanics among runners with different FSPs have shown difference in IFMs in transmitting ground reaction force and lever the arms to propel the body during running [13, 14]. Unfortunately, no investigation has compared IFM morphology and isometric strength among runners with different strike patterns and the relationship between morphology and isometric strength. Therefore, the present study aims to explore and compare IFMs morphology and isometric strength between habitual RFS and FFS runners and then to investigate correlation between morphology and strength. We hypothesise that FFS runners would have greater IFMs morphology and isometric strength than RFS pattern and plantar IFM morphology correlated with isometric flexor strength.

## Materials & methods

### Participants

A total of 70 healthy adult males with no diagnosed history of lower limb musculoskeletal injury or other medical problems in the previous six months volunteered to participate in the study [15, 16]. All participants were long-distance male runners and ran regularly for at least 10 miles per week [3]. Each participant was right-leg dominant (the dominant leg was defined as one’s preferred leg when kicking a ball). A written informed consent was obtained from every participant before the experiments, and this study was approved by the Human Ethics Committee of the Shanghai University of Sport (102772021RT130).

### Data collection

Before the test, participants’ height and body mass were measured. We recorded their self-reported running age. A participant’s FSP was verified utilising a video camera according to published articles [17, 18]. Owing to the low occurrence rates of MFS and FFS, we grouped MFS and FFS as non-rearfoot strike (NRFS) [15, 16, 19].

The participants were asked to lie and place their dominant ankle in the neutral position. The morphological properties of the IFMs were assessed with an ultrasound device (Diagnostic Ultrasound System, M7 Super, Mindary, China), which was verified to be reliable in quantifying muscle structures and better understand their contributions to foot function [20]. After applied ultrasound gel on the head of an ML6-15-D probe (10 MHz maximum frequency), we measure the morphological properties of the IFMs according to our previous studies [21]: 1) place the probe at the medial calcaneal tuberosity toward the navicular tuberosity to measure the thickest part of ABH, 2) align the probe longitudinally on the line from the medial tubercle of the calcaneus to the third toe to scan the thickest part of FDB, 3) place the probe longitudinally along the muscle fibers at the talocalcaneonavicular joint to locate the thickest part of QP, 4) marked the first metatarsal, and then place the probe longitudinally along the shaft to capture the thickest part of the FHB, 5) marked 50% of the line connecting fibular head and the inferior border of the lateral malleolus, place the probe longitudinally to capture thickest part of the peroneus longus and brevis (PER), 6) place the probe longitudinally in front of the calf over 20% of the distance between the fibular head and the inferior border of the lateral malleolus to obtain thickest part of the tibialis anterior (TA). For the CSA of selected muscles, based on the location of selected muscles thickness, we rotated probe at 90° to obtain cross-sectional images. Due to the scanning range of the probe, the CSA of the TA cannot be captured completely. Three pictures of each muscle were obtained for statistical analysis. The detailed testing protocol have been published in our previously measurement protocol [21].

In the IFMs isometric strength test, micro hand-held dynamometry (Hoggan health industries, Draper, UT, USA) with a digital reading of peak force ranging from 3.6 N to 1334.5 N in 0.4N increments was utilised [22, 23]. This tool has been verified to have good to excellent reliability and validity for most measures of isometric lower limb strength and power in a healthy population, particularly for proximal muscle groups [22–25]. After participants performed a 5-minute warm-up on a treadmill at a comfortable and self-selected pace, they were instructed to sit in a chair with 90° flexion of the knee and neutral ankle position to performed the IFMs isometric strength test. Doming test, also known as the short-foot exercise, we placed the dynamometer against the navicular tuberosity, and then instruct the participant to slide the forefoot toward the heel and raise the arch as much as possible without lifting or curling the toes. For the toe flexion strength test (Toe1, Toe2345, Toe12345), the dynamometer was fixed to the front side of the wooden frame, connect the toe to the dynamometer by carabiner, then the participants were encouraged to remain stable and pull as hard as possible for 3 s and then relax the grip according to our published studies [21]. Three successful muscle strength test results were averaged for further statistical analysis.

### Data reduction and statistical analysis

LabVIEW software was used in analysing the original strength data (CSV files). For the toe flexion strength (Toe1, Toe2345, Toe12345), the automatic calculation option in the calculation list was used in calculating the peak strength. As for the doming strength, the manual calculation option was selected, then the movable 0.5 s window in the shape of a plateau was manually adjusted for the calculation of the average force. To minimise the effects of different body built, IFMs morphology and isometric strength were all normalised by body weight [26].

All dependent variables were presented as mean and standard deviation (SD). The Kolmogorov–Smirnov test was used to test the normal distribution. Independent T-tests was used to compare IFM morphology and isometric strength in runners with different foot strike patterns. We then calculated 95% confidence interval (CI) and Cohen’s d. Pearson correlation were used in calculating the correlation between the cross-sectional area of IFM and isometric strength [27]. With regard to the correlation coefficient, |r| < 0.3 implies weak correlation, 0.3 ≤ |r| < 0.5 suggests low correlation, 0.5 ≤|r| < 0.8 denotes moderate correlation and |r| ≥0.8 indicates high correlation. Significance was set at alpha of <0.05. All statistics were performed using SPSS 22 software (IBM, Armonk, NY).

## Results

Of the participants included in our study, 32 participants were verified as habitual RFS (age of 21.8 ± 2.4 years; body height of 177.0 ± 5.5 cm; body mass of 69.8 ± 10.5 kg; running age of 8.0 ± 3.3 years; weekly running mileage of 13.3 ± 6.0 km), and 38 participants were verified as habitual NRFS (age of 20.9 ± 1.7 years; body height of 178.9 ± 6.6 cm; body mass of 73.6 ± 7.9; running age of 5.4 ± 3.8 years; weekly running mileage of 19.7 ± 14.4 km).

On the thickness and cross-sectional area of IFMs, although no significant difference was found for the ABH, FDB, QP and FHB, we found RFS runners had significantly thicker tibialis anterior (*p* = 0.01, ES = 0.64, 95% CI [0.01–0.07]) (Table 1). The results of the muscle strength test revealed that RFS runners had significant higher toe flexion strength in Toe2345 (*p* = 0.04, ES = 0.50, 95% CI [0.01–0.27]). No significant differences in Toe1, Toe12345 and doming test were found (Table 2).

**Table 1:** Comparison of the IFMs morphology of RFS and NRFS runners.

		RFS	NRFS	P values	Cohen’s D	95% confidence interval	
Thickness (mm/kg)	ABH	0.19±0.03	0.19±0.03	0.39	-0.21	-0.02	0.01
	FDB	0.15±0.03	0.14±0.02	0.49	0.17	-0.01	0.02
	QP	0.12±0.03	0.12±0.02	0.42	0.21	-0.01	0.02
	FHB	0.23±0.03	0.22±0.04	0.28	0.24	-0.01	0.03
	TA	0.42±0.06	0.38±0.07	**0.01**	0.64	0.01	0.07
	PER	0.25±0.06	0.22±0.04	0.08	0.42	0.00	0.05
Cross-sectional area(cm^2^/kg)	ABH	0.04±0.01	0.04±0.01	0.87	0.00	0.00	0.00
	FDB	0.04±0.01	0.03±0.01	0.30	0.29	0.00	0.01
	QP	0.03±0.01	0.03±0.01	0.56	-0.12	-0.01	0.00
	FHB	0.05±0.01	0.05±0.01	0.24	0.30	0.00	0.01
	PER	0.08±0.03	0.08±0.03	0.38	0.21	-0.01	0.02

Note: Values are means ± standard deviation (SD); significant differences (P < 0.05) are highlighted in bold. Abductor halluces (ABH), flexor digitorum brevis (FDB), quadratus plantae (QP), flexor hallucis brevis (FHB), tibialis anterior (TA), peroneus longus and brevis (PER).

**Table 2:** Comparison of the IFMs isometric strength of RFS and NRFS runners.

	RFS	NRFS	P values	Cohen’s D	95% confidence interval	
Toe1(N/kg)	1.19±0.27	1.07±0.28	0.07	0.44	-0.01	0.25
Toe2345(N/kg)	1.18±0.29	1.04±0.26	**0.04**	0.50	0.01	0.27
Toe12345(N/kg)	1.40±0.32	1.26±0.27	0.07	0.43	-0.01	0.27
Doming(N/kg)	1.52±0.44	1.48±0.31	0.70	0.09	-0.14	0.21

Note: Values are means ± standard deviation (SD); significant differences (P < 0.05) are highlighted in bold.

**Fig 1:**
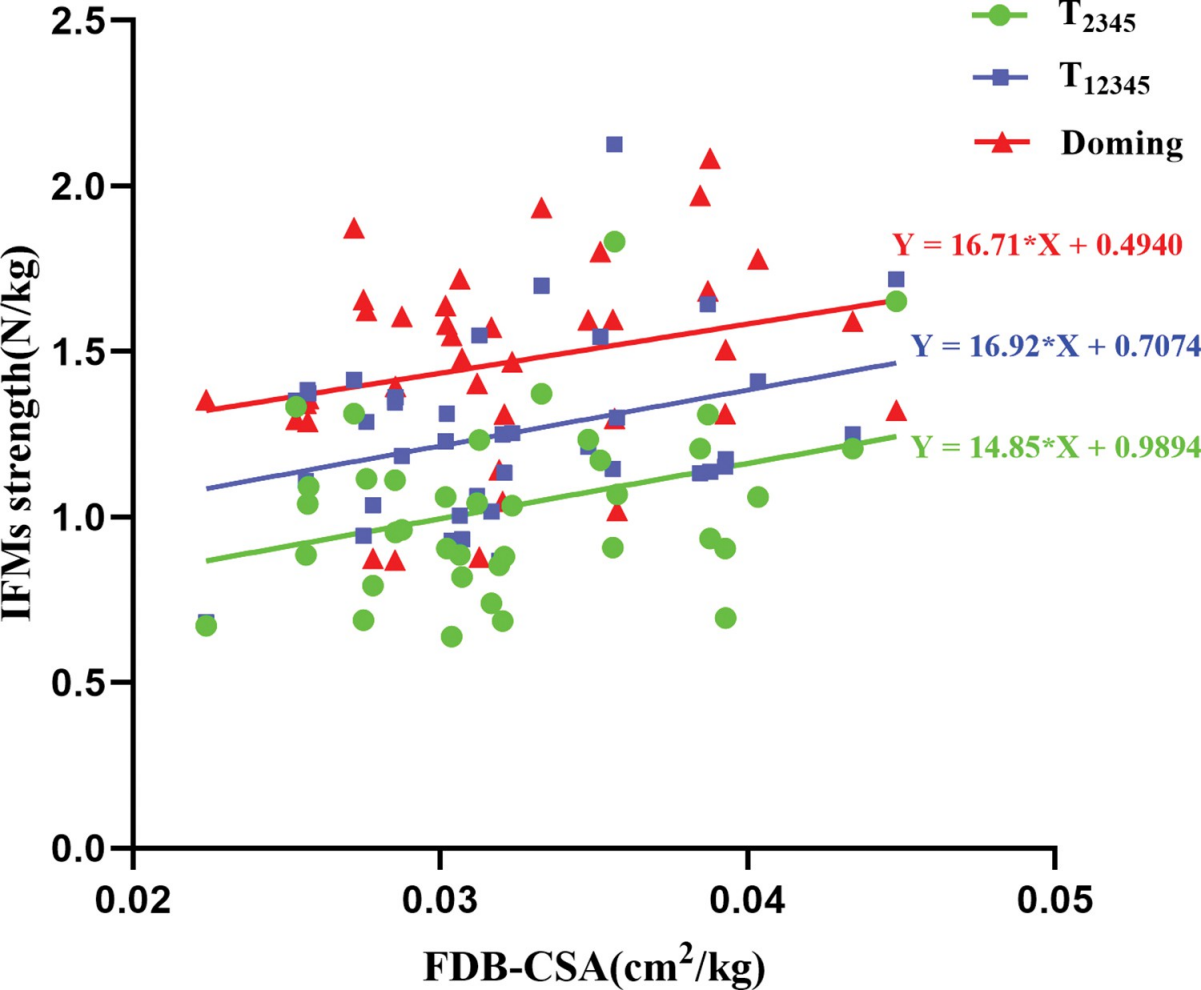
Correlation coefficient of FDB CSA and IFMs strength for NRFS runners. FDB: flexor hallucis brevis; CSA: cross-sectional area; IFMs: intrinsic foot muscles; T_2345_: Toe2345 flexion strength; T_12345_: Toe12345 flexion strength.

No significant correlation was found between the IFMs cross-sectional area and isometric strength in RFS runners. However, in runners with NRFS patterns, the cross-sectional area of the FDB was positively correlated with Toe2345 flexion strength (*r* = 0.33, *p* = 0.04), Toe12345 (*r* = 0.37, *p* = 0.02) and doming test (*r* = 0.36, *p* = 0.03; Fig 1; Table 3).

**Table 3:** Correlation coefficient of IFMs cross-sectional area and isometric strength.

Muscle	Muscle	RFS		NRFS	
strength (N/kg)	morphology (cm^2^/kg)	R	P values	R	P values
Toe_1_	ABH	-0.17	0.35	0.32	0.05
	FHB	0.23	0.21	0.15	0.38
Toe_2345_	FDB	0.18	0.33	0.33	**0.04**
	QP	0.08	0.68	-0.10	0.54
Toe_12345_	ABH	-0.11	0.53	0.15	0.37
	FHB	-0.07	0.72	0.04	0.81
	FDB	0.30	0.10	0.37	**0.02**
	QP	0.20	0.30	-0.11	0.49
Doming	ABH	-0.25	0.16	0.00	0.10
	FHB	0.31	0.09	0.07	0.66
	FDB	0.07	0.70	0.36	**0.03**
	QP	0.05	0.79	-0.08	0.63

Note: Values are means ± standard deviation (SD); significant differences (P < 0.05) are highlighted in bold. Abductor halluces (ABH), flexor digitorum brevis (FDB), quadratus plantae (QP), flexor hallucis brevis (FHB), tibialis anterior (TA), peroneus longus and brevis (PER).

## Discussion

The purpose of this study was to compare IFM morphology and isometric strength between habitual RFS and NRFS runners and then to investigate the correlation between morphology and isometric strength. In partial support of the hypothesis, our study found IFMs morphology and isometric strength are correlated and associated with foot strike pattern.

In the current study, although we did not observe any difference in plantar muscles, we found that the tibialis anterior in RFS runners was significantly thicker than that in NRFS runners. Given that the ankle was in dorsiflexion when the heel comes into contact with the ground in RFS runners [5], the EMG amplitudes of the dorsiflexor muscles, such as the tibialis anterior, were higher to absorb impact force than those in NRFS runners [6], the biomechanical characteristics of RFS runners may explain their thicker tibialis anterior. As for other anatomy parameters, our findings were inconsistent with those presented by Gonzales et al [3], who reported no differences in the fascicle length, cross-sectional area and subcutaneous fat thickness of the tibialis anterior among runners with different foot strike patterns. On plantar muscle morphology, the thickness and cross-sectional area of IFMs were smaller than those of the thigh and calf muscles, and the IFMs were covered by plantar fascia, which prevented ultrasound devices from detecting slight changes in foot muscles [9, 28]. Further, Taddei et al [29, 30] proposed that the ABH, FDB and FHB have various origins and insertions, and four layers of IFMs as local stabilisers increase the difficulty of distinguishing them.

Although hand-held dynamometry was verified to have high reliability and validity in assessing IFM strength [31–33], few studies have explored difference in IFM isometric strength among runners with different foot strike patterns. Given that FFS runners have a higher mean MVC plantarflexion strength than RFS runners in stochastic cluster analysis [34], FFS runners have significantly lower RMS activity in the tibialis anterior during the terminal swing phase and significantly greater RMS activity in the terminal swing phase [6]. Hence, we speculated that NRFS runners have larger IFM isometric strength than RFS runners. Unfortunately, the results of IFM isometric strength seem to contradict our research hypothesis. We found that RFS runners had significant higher toe flexion strength in Toe2345 than NRFS runners. A possible interpretation for this conflicting result is that NRFS runners mainly absorb and buffer a larger ground reaction force through their stronger calf muscles when their feet touch the ground, relatively small adaptation in IFMs seem insufficient to result in significant difference. For RFS runners, the strength of the tibialis anterior muscle is insufficient, runners need IFMs to produce propulsion force, push the body forward and increase the spring effect of the foot arch [12, 35, 36]. Owing to the absence of related studies, this assumption still needs to be confirmed by more rigorous study in the future.

According to the results of correlation between IFMs cross-sectional area and toe flexor strength, we speculated FDB may act as an important muscle positively correlated to Toe2345, Toe12345 and Doming among NRFS runners when their feet touch on the ground. The results are consistent with previous studies, which have reported that foot morphological characteristics can effectively predict foot muscle strength [37] and suggested the use of specific foot muscle training sessions to improve foot ability and prevent foot injury. The FDB is the most superficial among the plantar muscles [8, 11], and thus it has become the main focus in the fields of sports science and rehabilitation. Jacob et al [38] combined anthropometrical and plantar pressure data and revealed that FDB muscles can exert a force approximately 13% of body weight during the propulsive phase of walking. A similar EMG study has reported a small amount of activity in abductor hallucis and FDB during relaxed standing and significant increase in activity at increased postural demands [39]. Mann et al [40] demonstrated that the abductor hallucis and FDB are active during the stance phase of gait and continued until toe off. Our previous systematic review and meta-analysis indicated that IFM training can exert positive biomechanical effects on the medial longitudinal arch and improve dynamic postural balance. Combing with the results of correlation between FDB size and strength among NRFS runners in the current study, we suggest that sports enthusiasts, especially NRFS runners, could adopt IFM strength exercises to increase the function of FDB shock absorbers and locomotive effectors.

The present study has several limitations. First, owing to the scanning range of the probe, the CSA of the TA cannot be captured completely. Second, although the participants were instructed to sit in standard position during isometric strength test, which seem hardly to eliminate the contribution of extrinsic muscles in the plantar region. Additionally, only male population was included in our study, future studies should also employ female and utilizing advanced technology to gain more insights into the musculature and strength of the foot.

## Conclusions

IFMs morphology and isometric strength were associated with foot strike pattern, preliminary findings provide new perspectives for NRFS runners through the simple measurement of IFMs morphological characteristics predicting IFMs strength, future studies could adopt IFMs training to compensate the muscle strength defects and prevent foot-related injuries.

## Supporting information

S1 File Raw data of main figures and tables used in this study. (XLSX)
